# Finite-Length Analysis for Spatially Coupled LDPC Codes Based on Base Matrix

**DOI:** 10.3390/e25071041

**Published:** 2023-07-11

**Authors:** Yang Liu, Sha Sun, Yuzhi Zhang, Bin Wang

**Affiliations:** School of Communication and Information Engineering, Xi’an University of Science and Technology, Xi’an 710054, China

**Keywords:** spatially coupled LDPC codes, finite-length performance analysis, peeling decoder

## Abstract

Spatially coupled low density parity check (SC-LDPC) are prominent candidates for future communication standards due to their “threshold saturation” properties. To evaluate the finite-length performance of SC-LDPC codes, a general and efficient finite-length analysis from the perspective of the base matrix is proposed. We analyze the evolution of the residual graphs resulting at each iteration during the decoding process based on the base matrix and then derive the expression for the error probability. To verify the effectiveness of the proposed finite-length analysis, we consider the SC-LDPC code ensembles constructed by parallelly connecting multiple chains (PC-MSC-LDPC). The analysis results show that the predicted error probabilities obtained by using the derived expression for the error probability match the simulated error probabilities. The proposed finite-length analysis provides a useful engineering tool for practical SC-LDPC code design and for analyzing the effects of the code parameters on the performances.

## 1. Introduction

Spatially coupled low-density parity check (SC-LDPC) codes have been proven to improve the belief propagation (BP) thresholds up to the maximum a posterior (MAP) thresholds of the underlying LDPC block codes for the binary erasure channel (BEC) [1]. Afterwards, many new structures were proposed to achieve better thresholds or low-complexity/delay decoding, including designing the coupling pattern, eliminating small absorbing/trapping sets, introducing slight irregularities and so on [2,3,4]. In addition, most of the literature focused on applying the concept of spatial coupling on other error correction codes to improve the decoding thresholds, such as spatially coupled repeat-accumulate (SC-RA) codes, spatially coupled turbo codes (SC-TCs), spatially coupled precoded rateless codes and so on [5,6,7]. Moreover, spatial coupling need not be limited to forming a single chain, and more general structures formed by connecting multiple coupled chains were presented to improve the decoding thresholds [8,9,10]. Different from connecting multiple identical coupled chains, the SC-LDPC codes constructed by parallelly connecting multiple different chains (PC-MSC-LDPC) were proposed in [11], which showed that the thresholds of the PC-MSC-LDPC code ensembles with flexible rates are very close to Shannon limits over the BEC.

The studies stated above mainly focused on the asymptotic performance analysis of the SC-LDPC ensembles. To analyze the finite-length performances, a scaling law for predicting the error probability of the SC-LDPC codes over the BEC using the peeling decoder (PD) was proposed in [12], which extended the finite-length analysis for LDPC codes in [13]. At each iteration, if the variable node is not erased through the channel or connected to a degree-one check node, it will be recovered successfully and then removed from the decoding graph along with all its attached edges. This decoding process gives rise to a sequence of residual graphs. Therefore, the analysis of the PD process is equivalent to analyzing the evolution of the residual graphs, which can be transformed to analyze the evolution of the degree distributions (DDs) on the residual graphs. In [13], it was pointed out that the DDs on the residual graphs at any time converge to a multivariate Gaussian. As a result, by computing the mean and the variance of the DDs evolution during the PD process, the error probability can be estimated. Following this principle, a finite-length analysis for the loop ensemble constructed by connecting two identical coupled chains was presented in [14], which showed that connecting two coupled chains can result in better thresholds and improved finite-length performances.

These finite-length analyses focused on particular code structures, either the single SC-LDPC code ensemble or the loop ensemble. When the code structure is changed, these analyses will be either very complicated or invalid. According to the working principle of PD, it can be seen that the core of the finite-length analysis over the BEC is analyzing the DDs evolution of variable nodes and check nodes on the residual graph. The essence is to determine whether one variable/check node on the residual graph is removed or not according to the erasure probabilities computed by the received messages from the connected edges, which depends on the interconnections between the variable nodes and check nodes. Inspired by this, we proposed a general finite-length performance analysis from the perspective of the base matrix for SC-LDPC codes. We considered embedding the base matrix into analyzing the residual graph evolution during the decoding process. In particular, based on the base matrix, we derived the mean graph evolution and estimated the variance of the DDs on the residual graphs at each iteration to predict the error probabilities. To verify the effectiveness of the proposed finite-length analysis, we considered the PC-MSC-LDPC codes because they have special connection structures that cannot be analyzed by the existing analyses. Using this expression, we plot the predicted error probabilities for different PC-MSC-LDPC codes and also show the error probabilities by simulations. The comparison results show that the predicted error probabilities can fit the simulated ones well. Since this analysis is performed on the base matrix, it can be applied to other spatially coupled code ensembles defined by the base matrix, including the single SC-LDPC code ensemble and the loop ensemble. The analysis results of the conventional SC-LDPC codes demonstrate this statement.

This paper is organized as follows. In Section 2, we describe the SC-LDPC and PC-MSC-LDPC code ensembles using the base matrix. In Section 3, we analyze the graph evolution based on the base matrix, including the mean graph evolution and the variance estimation. In Section 4, we show and compare the results for different PC-MSC-LDPC codes and the conventional SC-LDPC codes. In Section 5, we conclude our work.

## 2. Construction of PC-MSC-LDPC Codes

### 2.1. SC-LDPC Codes

A (J,K,L) SC-LDPC coupled chain was constructed by coupling *L* disjoint and small (J,K)-regular LDPC protographs. Each protograph was placed at one position in order and each position was denoted by *u*, u=1,2,…,L. Here, we considered a conventional fully connected coupling pattern to couple these *L* protographs. Specifically, let w=gcd(J,K), which denotes the greatest common divisor of *J* and *K*. Then, there are $J^{\prime }$ check nodes and $K^{\prime }$ variable nodes at each position with $J^{\prime }=J/w$ and $K^{\prime }=K/w$. To couple these *L* protographs, we spread *J* edges of each variable node at position *u* to all adjacent check nodes at position u+i, i=0,1,…,w−1. In turn, for each check node at position *u*, *K* edges will be connected to all nearby variable nodes at position u−i, i=0,1,…,w−1. To terminate the coupled chain, w−1 extra positions only including additional check nodes will be added at the end.

A (J,K,L) SC-LDPC coupled chain can be viewed as a protograph and its associated incidence matrix $B_{\mathrm{sc}}$, called a base matrix, is defined in (1), where the submatrices $B_{i}$, i=0,…,w−1 are identical $J^{\prime }\times K^{\prime }$ all-one matrices. A (J,K,L,M) SC-LDPC code can be obtained by taking an “*M-lifting*” of the (J,K,L) coupled chain [15]. Specifically, the parity check matrix can be generated by replacing each one entry by one M×M random permutation matrix and each zero entry by one M×M all-zero matrix.

(1) $$\begin{array}{c} B_{\mathrm{sc}}={\left[\;\;\;\begin{array}{cccc} B_{0} & & & \\ B_{1} & B_{0} & & \\ \vdots & B_{1} & \ddots & \\ B_{w-1} & \vdots & & B_{0}\\ & B_{w-1} & \ddots & B_{1}\\ & & & \vdots \\ & & & B_{w-1} \end{array}\;\;\;\right]}_{J^{\prime }\left(L+w-1\right)\times K^{\prime }L}. \end{array}$$

### 2.2. PC-MSC-LDPC Codes

Consider *C* independent and unconnected coupled chains with the same coupling length *L*, where each chain has a different rate. The *k*th chain is denoted as $B(J_{k},K_{k},L)$ and there are $J_{k}^{\prime }$ check nodes and $K_{k}^{\prime }$ variable nodes at each position, where $J_{k}^{\prime }=J_{k}/w_{k}$, $K_{k}^{\prime }=K_{k}/w_{k}$, $w_{k}=\gcd\left(J_{k},K_{k}\right)$ and k=1,2,…,C. The base matrix is denoted as $B_{\mathrm{sc},k}$ and the size is $J_{k}^{\prime }\left(L+w_{k}-1\right)\times K_{k}^{\prime }L$. Let $a=\min\{K_{1}^{\prime },K_{2}^{\prime },\ldots ,K_{C}^{\prime }\}$ and $b=\min\{J_{1}^{\prime },J_{2}^{\prime },\ldots ,J_{C}^{\prime }\}$.

**Figure 1:**
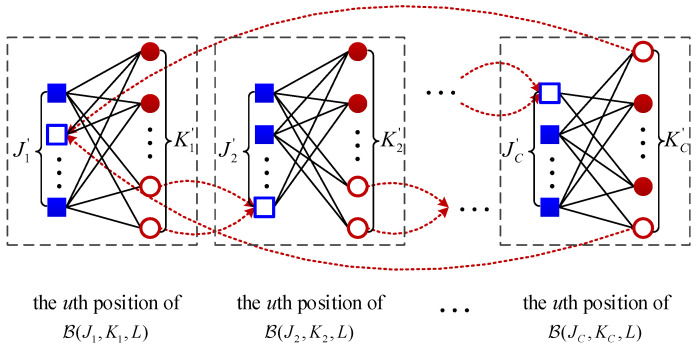
The connection structure at the *u*th position of the code ensemble $P(J_{1},K_{1},\ldots ,J_{C},K_{C},L)$.

Then, connect these *C* chains parallelly by edge exchanges. Specifically, *a* variable nodes and *b* check nodes are randomly selected for each position *u* of every chain $B(J_{k},K_{k},L)$, where u=1,2,…,L and k=1,2,…,C. Next, break the edges between these selected *a* variable nodes and *b* check nodes at each position *u* of $B(J_{k},K_{k},L)$ and simultaneously connect these broken edges to *b* check nodes at each position *u* of $B(J_{z},K_{z},L)$ with z=(kmodC)+1. The construction process starts from k=1 and stops until k=C. Take the *u*th position to illustrate this process in Figure 1, where the blue blank squares and red blank circles are the selected check nodes and variable nodes, respectively, and the red dash lines represent the exchange edges between two adjacent chains. The base matrix is

(2) $$\begin{array}{c} \;\;B_{\mathrm{pc}}={\left[\begin{array}{ccccc} B_{\mathrm{sc},1}^{{}^{\prime }} & & & & L_{C}\\ L_{1} & B_{\mathrm{sc},2}^{{}^{\prime }} & & & \\ & L_{2} & \ddots & & \\ & & \ddots & B_{\mathrm{sc},C-1}^{{}^{\prime }} & \\ & & & L_{C-1} & B_{\mathrm{sc},C}^{{}^{\prime }} \end{array}\right]}_{m\times n}, \end{array}$$

where $B_{\mathrm{sc},k}^{{}^{\prime }}$ denotes the remaining matrix after removing the exchange edges from the base matrix $B_{\mathrm{sc},k}$ and $L_{k}$ represents the interconnections between the chain $B(J_{k},K_{k},L)$ and $B(J_{z},K_{z},L)$, k=1,2,…,C and z=(kmodC)+1. The size of $L_{k}$ is $J_{z}^{\prime }\left(L+w_{z}-1\right)\times K_{k}^{\prime }L$. $m=\sum_{k=1}^{C}J_{k}^{\prime }\left(L+w_{k}-1\right)$ and $n=\sum_{k=1}^{C}K_{k}^{\prime }L$. Denote the PC-MSC-LDPC code ensemble defined by this base matrix as $P(J_{1},K_{1},\ldots ,J_{C},K_{C},L)$. Take an “*M-lifting*” operation on this base matrix and obtain the parity check matrix of a PC-MSC-LDPC code. Denote the PC-MSC-LDPC code as $C(J_{1},K_{1},\ldots ,J_{C},K_{C},L,M)$. More details about the asymptotic performance analysis can be found in [11].

**Figure 2:**
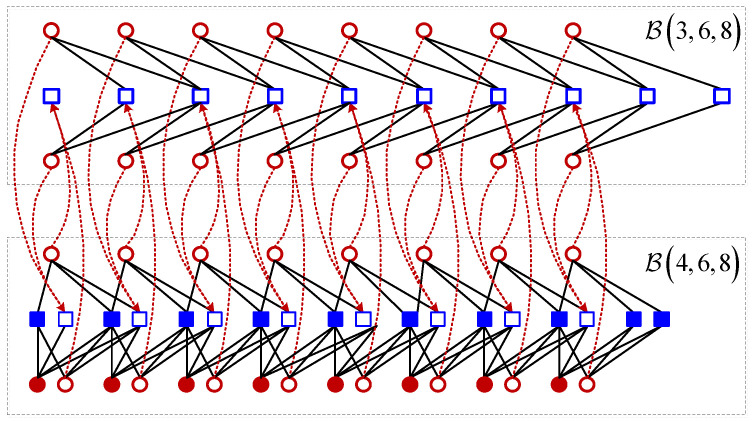
The connection structure of two chains B(3,6,8) and B(4,6,8) in parallel.

Example: Consider two coupled chains: B(3,6,8) and B(4,6,8). Since $K_{1}^{\prime }=2$, $J_{1}^{\prime }=1$ for B(3,6,8) and $K_{2}^{\prime }=3$, $J_{2}^{\prime }=2$ for B(4,6,8), we have a=2 and b=1. Thus, we need to select two variable nodes and one check node at each position for B(4,6,8). The connection structure of these two chains in parallel is shown in Figure 2, where the blue blank squares denote the selected one check node and the red blank circles are the selected two variable nodes. Specifically, at each position, we break the two edges between two variable nodes and one check node of B(3,6,8) and connect them to one selected check node of B(4,6,8) at the same position (shown in dash lines). Then, we break the two edges between these two selected variable nodes and one selected check node of B(4,6,8), and connect them to one check node of B(3,6,8) (shown in red dash lines). The base matrix can be obtained by (2).

## 3. Graph Evolution under Peeling Decoder

Following the PD working principle, we proposed a general finite-length analysis based on the base matrix to predict the error probabilities. Without a loss of generality, we considered the PC-MSC-LDPC codes transmitted over the BEC with erasure probability ϵ under PD.

### 3.1. Denotations of DDs

As described in Section 2, denote the check node at the *i*th row of the base matrix B as Type-*i* check node and the variable node at *j*th column as Type-*j* variable node, where 1≤i≤m and 1≤j≤n. The number of each type of check/variable node is *M*. The degrees of Type-*i* check node and Type-*j* variable node are ${\mathrm{dc}}_{i}=\sum_{j=1}^{n}B(i,j)$ and ${\mathrm{dv}}_{j}=\sum_{i=1}^{m}B(i,j)$, respectively, where B(i,j) is the entry at the *i*th row and *j*th column of B.

To describe the graph evolution under PD, let *l* denote time and let it be normalized by τ=l/M. Since the PD peels off one variable node at each iteration from the decoding graph and there are ϵMn variable nodes in total at the start of the decoding process, ϵMn iterations are required on average in order to reach the empty graph and realize successful decoding, i.e., τ∈[0,ϵn].

Let $V_{j}\left(l\right)$ denote the number of the remaining Type-*j* variable nodes and $R_{s,i}\left(l\right)$ denote the number of edges connected to Type-*i* check nodes with degree *s* at time *l*, where 1≤j≤n, $1\leq s\leq {\mathrm{dc}}_{i}$, 1≤i≤m. $V_{j}\left(l\right)$ and $R_{s,i}\left(l\right)$ are defined as the DDs at time *l*.

Denote $v_{j}\left(\tau \right)$ and $r_{s,i}\left(\tau \right)$ as the normalized versions, where they can be obtained by normalizing $V_{j}\left(l\right)$ and $R_{s,i}\left(l\right)$ with *M* at normalized time τ.

(3) $$v_{j}\left(\tau \right)=\frac{V_{j}\left(l\right)}{M},\;\;r_{s,i}\left(\tau \right)=\frac{R_{s,i}\left(l\right)}{M}.$$

Since the expected values are required during the graph evolution, denote the expected values of $v_{j}\left(\tau \right)$ and $r_{s,i}\left(\tau \right)$ as ${\hat{v}}_{j}\left(\tau \right)=E\left[v_{j}\left(\tau \right)\right]$ and ${\hat{r}}_{s,i}=E\left[r_{s,i}\left(\tau \right)\right]$.

### 3.2. Mean Graph Evolution

Initialization Step: The number of the correctly received Type-*j* variable nodes is (1−ϵ)M after passing the BEC with erasure probability ϵ. At l=0, PD removes all these correctly received variable nodes along with their attached edges from the decoding graph. The expected number of Type-*j* variable nodes is given by

(4) $$E[V_{j}\left(0\right)]=\epsilon M,1\leq j\leq n,$$

and the normalized version is ${\hat{v}}_{j}\left(0\right)=E\left[v_{j}\left(0\right)\right]=E\left[V_{j}\left(0\right)\right]/M$.

At l=0, since PD removed all correctly received variable nodes along with their attached edges from the decoding graph, the check nodes on the residual graph will lose edges and the degree will be decreased. If the degree of a Type-*i* check node with degree ${\mathrm{dc}}_{i}$ is decreased to *s*, it means that a total of ${\mathrm{dc}}_{i}-s$ edges of this check node are connected to the correctly received variable nodes. Therefore, the expected value of $R_{s,i}\left(0\right)$ is

(5) $$E\left[R_{s,i}\left(0\right)\right]=sM\left(\begin{array}{c} {\mathrm{dc}}_{i}\\ s \end{array}\right)\epsilon^{s}{\left(1-\epsilon \right)}^{{\mathrm{dc}}_{i}-s},1\leq i\leq m.$$

At the right side of Equation (5), the former part sM represents the total number of edges connected to Type-*i* check nodes with degree *s* and the latter part is the probability that the degree of a Type-*i* check node is *s*. The normalized version is ${\hat{r}}_{s,i}\left(0\right)=E\left[r_{s,i}\left(0\right)\right]=E\left[R_{s,i}\left(0\right)\right]/M$.

Evolution Step: At time *l*, one degree-one check node is randomly selected and then removed along with its connected variable node and all connected edges. A new residual graph is produced.

The mean graph evolution is determined by the expected values of $r_{r,i}\left(\tau \right)$ and $v_{j}\left(\tau \right)$, which can be obtained by solving the following differential equations:

(6) $$\frac{\partial {\hat{v}}_{j}\left(\tau \right)}{\partial \tau }=\frac{\partial E[v_{j}\left(\tau \right)]}{\partial \tau }=\frac{\partial E[V_{j}\left(l\right)]}{\partial l}=E\left[V_{j}\left(l+1\right)-V_{j}\left(l\right)\left|R_{q,p}\left(l\right),V_{t}\left(l\right),\forall q,p,t\right.\right],$$

(7) $$\frac{\partial {\hat{r}}_{s,i}\left(\tau \right)}{\partial \tau }=\frac{\partial E[r_{s,i}\left(\tau \right)]}{\partial \tau }=\frac{\partial E[R_{s,i}\left(l\right)]}{\partial l}=E\left[R_{s,i}\left(l+1\right)-R_{s,i}\left(l\right)\left|R_{q,p}\left(l\right),V_{t}\left(l\right),\forall q,p,t\right.\right],$$

where 1≤i≤m, $1\leq s\leq {\mathrm{dc}}_{i}$, 1≤j≤n, and they have unique solutions. As pointed out in [13], when M→∞, any samples of $v_{j}\left(\tau \right)$ and $r_{s,i}\left(\tau \right)$ follow ${\hat{v}}_{j}\left(\tau \right)$ and ${\hat{r}}_{s,i}\left(\tau \right)$ closely. The solutions of Equations (6) and (7) are given as follows.

Solution of Equation (6): At time *l*, assume the removed degree-one check node to be a Type-*c* check node, which is chosen randomly from all degree-one check nodes on the residual graph with uniform probability $p_{c}\left(l\right)$.

(8) $$p_{c}\left(l\right)=\frac{R_{1,c}\left(l\right)}{\sum_{i=1}^{m}R_{1,i}\left(l\right)}.$$

Then, the variable node connected to this removed Type-*c* degree-one check will be removed. Denote the probability that this variable node is a Type-*j* variable node as $\lambda_{c,j}\left(l\right)$.

(9) $$\lambda_{c,j}\left(l\right)=\frac{V_{j}\left(l\right)B_{\mathrm{pc}}\left(c,j\right)}{\sum_{u=1}^{n}V_{u}\left(l\right)B_{\mathrm{pc}}\left(c,u\right)},$$

where the denominator represents the total number of variable nodes connected to Type-*c* check nodes.

Since a Type-*j* variable node with probability $\lambda_{c,j}\left(l\right)$ is removed, the variation for the variable nodes is

(10) $$E\left[V_{j}\left(l+1\right)-V_{j}\left(l\right)|p_{c}\left(l\right)\right]=-\lambda_{c,j}\left(l\right).$$

Solution of Equation (7): At time *l*, if this Type-*j* variable node connected to the removed Type-*c* degree-one check node is removed, all the attached edges will be deleted, which results in every connected check node losing one edge. Then, denote the probability that a Type-*i* check node loses one edge as $\xi_{c,i}\left(l\right)$ and, specifically, $\xi_{c,c}\left(l\right)=1$.

(11) $$\xi_{c,i}\left(l\right)=\sum_{j=1}^{n}B_{\mathrm{pc}}\left(i,j\right)\lambda_{c,j}\left(l\right).$$

Since a Type-*c* check node with degree one and an edge connected to it are removed, the variation in the check nodes for the case i=c can be calculated as

(12) $$E\left[R_{s,c}\left(l+1\right)-R_{s,c}\left(l\right)|p_{c}\left(l\right)\right]=\left\{\begin{array}{c} \;\;-1,\;s=1\\ \;0,\;others \end{array}.\right.$$

For the case i≠c, the graph loses one edge with probability $\xi_{c,i}\left(l\right)$. This lost edge is connected to a degree-*s* check node with probability

(13) $$\frac{R_{s,i}\left(l\right)}{\sum_{q=1}^{{\mathrm{dc}}_{i}}R_{q,i}\left(l\right)}.$$

As a result, the graph will lose *s* edges of Type-*i* check nodes with degree *s* and gain s−1 edges of the Type-*i* check nodes with degree s−1. The expected graph evolution is

(14) $$E\left[R_{s,i}\left(l+1\right)-R_{s,i}\left(l\right)|p_{c}\left(l\right)\right]=s\xi_{c,i}\left(l\right)\frac{R_{s+1,i}\left(l\right)-R_{s,i}\left(l\right)}{\sum_{q=1}^{{\mathrm{dc}}_{i}}R_{q,i}\left(l\right)},\;\;$$

where $R_{s+1,i}\left(l\right)=0$ for $s={\mathrm{dc}}_{i}$.

Since the fraction of degree-one check nodes on the graph determines the successful decoding, we only consider the variation in the degree-one check nodes. In conclusion, by using Equations (10) and (14), the expected graph evolutions can be derived.

On the variable node side, we can obtain

(15) $$E\left[V_{j}\left(l+1\right)-V_{j}\left(l\right)\right]=-\sum_{p=1}^{m}\lambda_{c,j}\left(l\right)p_{c}\left(l\right).$$

On the degree-one check node side, we can obtain

(16) $$E\left[R_{1,i}\left(l+1\right)-R_{1,i}\left(l\right)\right]=-p_{c}\left(l\right)+\left(\frac{R_{2,i}\left(l\right)-R_{1,i}\left(l\right)}{\sum_{q=1}^{d_{c_{i}}}R_{q,i}\left(l\right)}\right)\left(p\left(l\right)\xi_{i}^{T}\left(l\right)-p_{i}\left(l\right)\right),$$

where $p\left(l\right)=[p_{1}\left(l\right)\;p_{2}\left(l\right)\;\cdot \cdot \cdot p_{m}\left(l\right)]$ and $\xi_{i}\left(l\right)=\left[\xi_{1,i}\left(l\right)\;\xi_{2,i}\left(l\right)\;\cdot \cdot \cdot \xi_{m,i}\left(l\right)\right]$.

Decoding Criteria: To ensure the successful decoding, the total number of degree-one check nodes must be kept positive until the whole graph is peeled off. Therefore, the BP threshold can be defined as the maximum ϵ to ensure that the mean fraction of degree-one check nodes ${\hat{r}}_{1}\left(\tau \right)$ is strictly positive for any τ∈[0,ϵn].

(17) $${\hat{r}}_{1}\left(\tau \right)=\sum_{i=1}^{m}{\hat{r}}_{1,i}\left(\tau \right)=\sum_{i=1}^{m}E[r_{1,i}\left(\tau \right)].$$

### 3.3. Variance Estimation

After describing the expected evolution of the random process $r_{1}\left(\tau \right)$, we need to compute the variance of $r_{1}\left(\tau \right)$ for estimating the error probability of the PC-MSC-LDPC codes. As pointed out in [12], for sufficiently large *M*, the distribution of $r_{1}\left(\tau \right)$ can converge to a Gaussian distribution with mean ${\hat{r}}_{1}\left(\tau \right)$ in Equation (17) and variance $\delta_{1}\left(\tau \right)$ in Equation (18). Therefore, we can estimate the variance empirically around the mean value using a large set of samples of $r_{1}\left(\tau \right)$ at time τ.

(18) $$\mathrm{Var}\left[r_{1}\left(\tau \right)\right]=E\left[{\left(r_{1}\left(\tau \right)-{\hat{r}}_{1}\left(\tau \right)\right)}^{2}\right]=\frac{1}{M}\sum_{i=1}^{m}\sum_{t=1}^{m}\delta_{1i,1t}=\frac{\delta_{1}\left(\tau \right)}{M}.$$

## 4. Performance Analysis and Results

**Figure 3:**
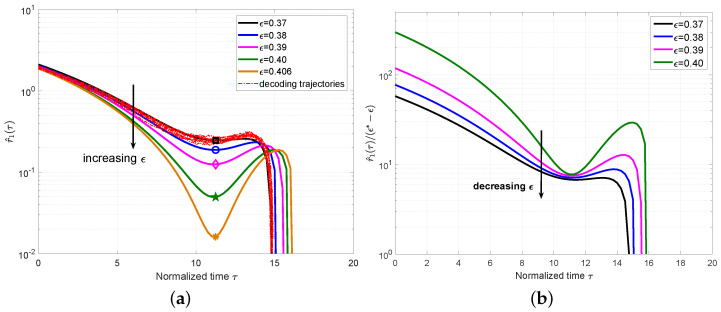
(**a**) Plot ${\hat{r}}_{1}\left(\tau \right)$ for the ensemble P(3,6,3,9,8) with M=500 and different ϵ. The decoding trajectories are included for ϵ=0.37. The symbols in each line correspond to the critical points for each ϵ. (**b**) Plot ${\hat{r}}_{1}\left(\tau \right)/(\epsilon^{*}$ − ϵ) with the threshold $\epsilon^{*}=0.4064$.

We first show the mean value ${\hat{r}}_{1}\left(\tau \right)$ for the PC-MSC-LDPC code ensemble P(3,6,3,9,8) with different ϵ and M=500 in Figure 3a. For ϵ=0.37, we also plot a set of 10 simulated decoding trajectories to confirm that they indeed concentrate around the predicted mean evolution. From Figure 3a, we can clearly observe that the local minimum decreases as ϵ increases and will be close to zero at the BP threshold $\epsilon^{*}=0.4064$ computed by the density evolution in [11], which also coincides with the definition of the BP threshold in Section 3.2. The time at this local minimum was defined as the *critical point* and denoted as $\tau^{*}$.

In order to associate the error probability with ϵ, we considered a first-order Taylor expansion around the threshold $\epsilon^{*}$ for ${\hat{r}}_{1}\left(\tau^{*}\right)$ at $\tau^{*}$.

(19) $${\hat{r}}_{1}\left(\tau^{*}\right)\approx {\hat{r}}_{1}\left(\tau^{*}\right){|}_{\epsilon^{*}}+\gamma \left(\epsilon^{*}-\epsilon \right)+O\left({\left(\epsilon^{*}-\epsilon \right)}^{2}\right).$$

We plot ${\hat{r}}_{1}\left(\tau \right)/\left(\epsilon^{*}-\epsilon \right)$ for the ensemble P(3,6,3,9,8) in Figure 3b. The approximately constant values can be observed for different ϵ at $\tau^{*}$, which indicates that it is reasonable to remove the high-order components in Equation (19). Using ${\hat{r}}_{1}\left(\tau^{*}\right){|}_{\epsilon^{*}=0}$, we can obtain $\gamma \approx {\hat{r}}_{1}\left(\tau^{*}\right)/\left(\epsilon^{*}-\epsilon \right)$, which can characterize the expected number of degree-one check nodes for a given ϵ at the critical point $\tau^{*}$.

**Figure 4:**
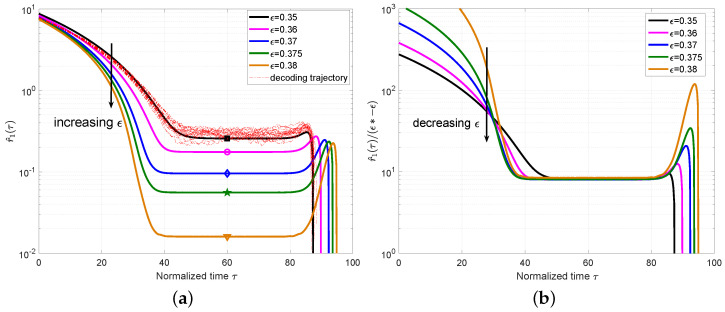
(**a**) Plot ${\hat{r}}_{1}\left(\tau \right)$ for the ensemble P(3,6,3,9,50) with M=500 and different ϵ. The decoding trajectories are included for ϵ=0.35. The symbols in each line correspond to the critical points for each ϵ. (**b**) Plot ${\hat{r}}_{1}\left(\tau \right)/(\epsilon^{*}$ − ϵ) with the threshold $\epsilon^{*}=0.3819$.

Then, we extended the analysis to the case of L=50 and plot the mean value ${\hat{r}}_{1}\left(\tau \right)$ of the ensemble P(3,6,3,9,50) with M=500 in Figure 4a, which also includes a set of 10 simulated decoding trajectories for ϵ=0.35 to verify the accuracy of the mean evolution. Different from the ensemble P(3,6,3,9,8), we can observe that the local minimum appears not only at one critical point but also for a period of time. This phase is named as a *steady phase* and the critical point $\tau^{*}$ can be any time during this phase. At this steady phase, the expected number of degree-one check nodes is almost constant, which confirms the conclusion in [1] that the decoding waves travel away from the boundaries toward the center of the coupled chain at a constant speed. We also plot ${\hat{r}}_{1}\left(\tau \right)/\left(\epsilon^{*}-\epsilon \right)$ for the ensemble P(3,6,3,9,50) in Figure 4b. The approximately constant values can be observed for different ϵ at $\tau^{*}$.

As pointed out in [12], the fraction of degree-one check nodes at $\tau^{*}$ dominates the code performance, so we only need to estimate the variance $\mathrm{Var}[r_{1}\left(\tau \right)]$ at $\tau^{*}$. Specifically, we produced a set of $10^{2}$ samples of $r_{1}\left(\tau \right)$ for each ϵ by using one randomly generated code from the PC-MSC-LDPC ensemble under the PD. For the ensemble P(3,6,3,9,8), the estimated $\delta_{1}\left(\tau^{*}\right)$ for different ϵ and *M* is listed in Table 1.

Since $r_{1}\left(\tau \right)$ converges to a Gaussian distribution [13], the error probability at $\tau^{*}$ can be obtained.

(20) $$P=Q\left(\frac{{\hat{r}}_{1}\left(\tau^{*}\right)}{\sqrt{\mathrm{Var}[{\hat{r}}_{1}\left(\tau^{*}\right)]}}\right)=Q\left(\frac{\gamma (\epsilon^{*}-\epsilon )}{\sqrt{\delta_{1}\left(\tau^{*}\right)/M}}\right).$$

**Figure 5:**
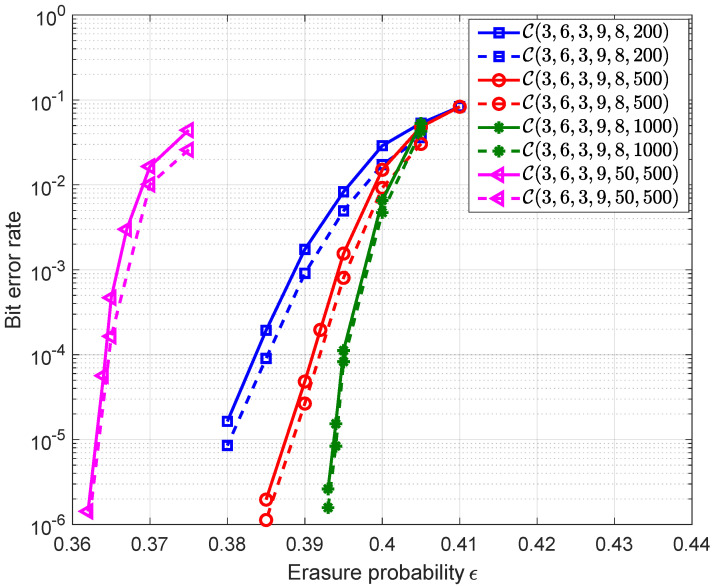
Simulated error probabilities (solid lines) and predicted error probabilities using the expression in Equation (20) (dash lines) for the different PC-MSC-LDPC codes. The rate and the codelength of C(3,6,3,9,50,500) are 0.584 and 125,000. The rate of the other three codes is 0.5 and the codelengths are 8000, 20,000 and 40,000 respectively.

Using Equation (20), we show the predicted error probabilities (dash lines) for different PC-MSC-LDPC codes in Figure 5 and also plot the simulated ones (solid lines) for comparisons. The results show that the predicted error probabilities are consistent with the simulated error probabilities but small gaps can be observed for relatively small *M*. They are caused due to two reasons. One is the deviation between the mean value ${\hat{r}}_{1}\left(\tau \right)$ and the true mean value of the process $r_{1}\left(\tau \right)$, but it deviates from the true mean value of the process $r_{1}\left(\tau \right)$ by less than $M^{-1/6}$. As M→∞, any sample of $r_{1}\left(\tau \right)$ follows ${\hat{r}}_{1}\left(\tau \right)$ closely. The other is the negligence of the decoding failure at $\tau \neq \tau^{*}$ caused by the small cycles or stopping sets in the graph. This effect is more severe for smaller values of *M*. However, since the SC-LDPC code ensemble has a linear growth of minimum distance with block length nM, the codes with small cycles or low-weight stopping sets can hardly be found for sufficiently large *M*. Therefore, when *M* increases to a few thousands, the effects on the prediction accuracy will be small enough to be ignored.

**Figure 6:**
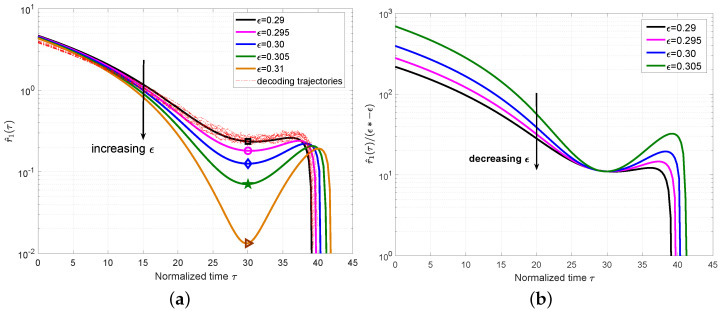
(**a**) Plot ${\hat{r}}_{1}\left(\tau \right)$ for the ensemble P(3,6,3,9,3,12,15) with M=200 and ϵ from 0.29 to 0.31. A set of 10 empirical trajectories for ϵ=0.29 are included. The symbols in each line correspond to the critical points for each ϵ. (**b**) Plot ${\hat{r}}_{1}\left(\tau \right)/(\epsilon^{*}$ − ϵ) with the threshold $\epsilon^{*}=0.3114$.

**Figure 7:**
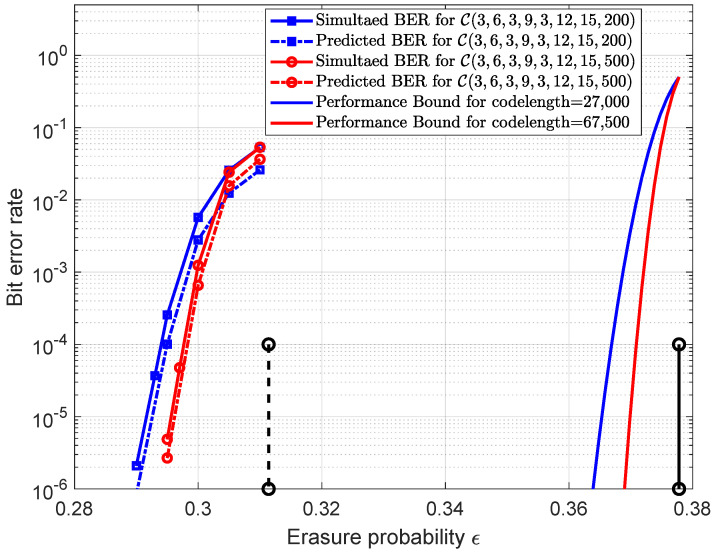
Simulated error probabilities (solid lines) and predicted error probabilities (dash lines) for the codes C(3,6,3,9,3,12,15,200) and C(3,6,3,9,3,12,15,500). The rate is 0.6222 and the codelengths are 27,000 and 67,500 respectively. The solid lines from left to right are the performance bounds for codelength = 27,000 and codelength = 67,500 in order. BP threshold (vertical dash line) and Shannon limit (vertical solid line) are also included.

Next, we extended the analysis to the case of connecting three different chains and considered the ensemble P(3,6,3,9,3,12,15). The mean value ${\hat{r}}_{1}\left(\tau \right)$ with M=200 and different ϵ is plotted in Figure 6. Similar results can be observed that, when approaching the threshold $\epsilon^{*}=0.3114$, the ${\hat{r}}_{1}\left(\tau^{*}\right)$ values gradually decrease to approximately zero. In Table 2, the $\delta_{1}\left(\tau^{*}\right)$ values are calculated for the codes generated from the ensemble P(3,6,3,9,3,12,15). The predicted error probabilities and the simulated ones for these codes are shown in Figure 7. The comparison results show that these two curves can match well and that the accuracy of the prediction gets better as *M* becomes larger. For comparison, the finite length performance bounds obtained by Equation (290) in [16] for different codelengths along with the BP threshold and Shannon limit are also plotted in Figure 7, from which we can observe that the gaps between the error probability curves and performance bounds are almost equal to the gap between the BP threshold and Shannon limit. It is known that the finite-length performance is consistent with the BP threshold. By increasing the coupling length, the BP threshold can be improved to be close to the Shannon limit, which can result in the error probabilities approaching the finite-length performance bounds.

**Figure 8:**
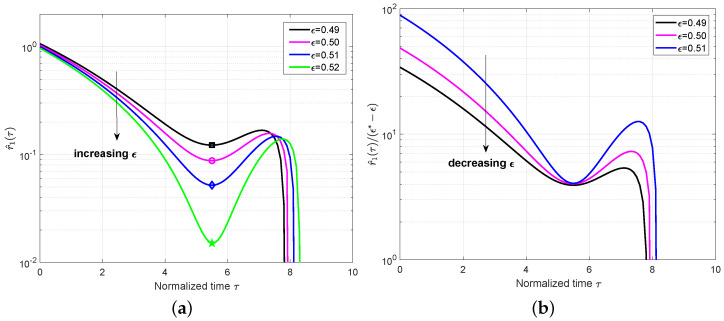
(**a**) Plot ${\hat{r}}_{1}\left(\tau \right)$ for the code C(3,6,8,700), where ϵ varies from 0.49 to 0.52. The symbols in each line correspond to the critical points for each ϵ. (**b**) Plot ${\hat{r}}_{1}\left(\tau \right)/(\epsilon^{*}$ − ϵ) with the BP threshold $\epsilon^{*}=0.5212$.

**Figure 9:**
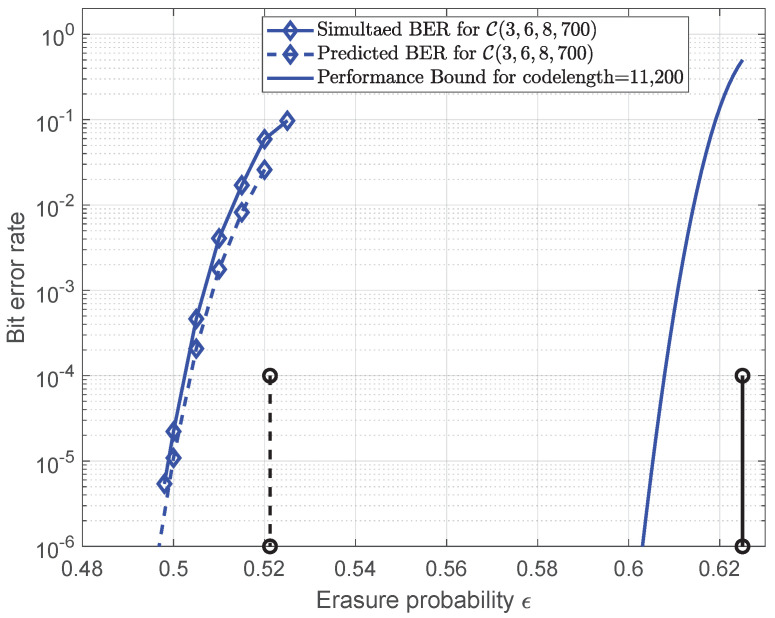
Simulated error probability and predicted error probability for the code C(3,6,8,700). The rate is 0.375 and the codelength is 11,200. The blue solid line is the performance bound for codelength=11,200. BP threshold (vertical dash line) and Shannon limit (vertical solid line) are also included.

Finally, we applied the analysis to the conventional SC-LDPC code C(3,6,8,700). The mean value ${\hat{r}}_{1}\left(\tau \right)$ with different ϵ and plot ${\hat{r}}_{1}\left(\tau \right)/\left(\epsilon^{*}-\epsilon \right)$ is shown in Figure 8. It can be observed that the local minimum values decrease when increasing ϵ to the BP threshold $\epsilon^{*}=0.5212$ and that they are small enough to be close to zero at ϵ=0.52. In addition, the approximately constant γ can be observed at the critical point as expected. Following similar steps, we computed and list the $\delta_{1}\left(\tau^{*}\right)$ values for different ϵ in Table 3. Using Equation (20), the predicted error probability for C(3,6,8,700) can be plotted in Figure 9. It was shown that the predicted performance using this error probability expression can fit well with the simulated performance, which can demonstrate the effectiveness of the proposed finite-length analysis for other SC-LDPC code ensembles.

## 5. Conclusions

This paper proposed a general finite-length analysis from the perspective of the base matrix over the BEC and applied it to the PC-MSC-LDPC code ensembles to verify the effectiveness. The results show that the predicted error probabilities obtained by using the derived error probability expression are consistent with the simulated error probabilities and that the accuracy of the prediction will be further improved when *M* increases. Since the proposed analysis is performed on the base matrix, it can be generalized to any spatially coupled ensembles defined by the base matrix, such as SC-RA codes and spatially coupled generalized LDPC codes. Finite-length performance analysis provides a useful engineering tool for practical code design and analyzing the effects of the code parameters on the performances.

## Figures and Tables

**Table 1:** The values of $\delta_{1}\left(\tau^{*}\right)$ for the ensemble P(3,6,3,9,8).

$\delta_{1}\left(\tau^{*}\right)$	ϵ=0.385	ϵ=0.39	ϵ=0.395	ϵ=0.40
*M* = 200	0.3526	0.3222	0.2548	0.1509
*M* = 500	0.5527	0.4796	0.4258	0.3031
$\delta_{1}\left(\tau^{*}\right)$	ϵ=0.393	ϵ=0.394	ϵ=0.395	ϵ=0.40
*M* = 1000	0.6174	0.6019	0.5978	0.4991

**Table 2:** The values of $\delta_{1}\left(\tau^{*}\right)$ for the ensemble P(3,6,3,9,3,12,15).

$\delta_{1}\left(\tau^{*}\right)$	ϵ=0.295	ϵ=0.30	ϵ=0.305	ϵ=0.31
*M* = 200	0.4777	0.4146	0.1988	0.0094
*M* = 500	0.7972	0.7711	0.5394	0.0275

**Table 3:** The values of $\delta_{1}\left(\tau^{*}\right)$ for the code C(3,6,8,700).

$\delta_{1}\left(\tau^{*}\right)$	ϵ=0.52	ϵ=0.515	ϵ=0.51	ϵ=0.505	ϵ=0.50	ϵ=0.495
*M* = 700	0.0422	0.1372	0.1694	0.2432	0.2825	0.3069

## Data Availability

Not applicable.

## Abbreviations

The following abbreviations are used in this manuscript:

SC-LDPC	Spatially coupled low-density parity check
BP	Belief propagation
MAP	Maximum a posterior
BEC	Binary erasure channel
SC-RA	spatially coupled repeat-accumulate
SC-TCs	spatially coupled turbo codes
PC-MSC-LDPC	Parallelly connecting multiple SC-LDPC
PD	Peeling decoder
DDs	Degree distributions
